# Ensembl Genomes 2016: more genomes, more complexity

**DOI:** 10.1093/nar/gkv1209

**Published:** 2015-11-17

**Authors:** Paul Julian Kersey, James E. Allen, Irina Armean, Sanjay Boddu, Bruce J. Bolt, Denise Carvalho-Silva, Mikkel Christensen, Paul Davis, Lee J. Falin, Christoph Grabmueller, Jay Humphrey, Arnaud Kerhornou, Julia Khobova, Naveen K. Aranganathan, Nicholas Langridge, Ernesto Lowy, Mark D. McDowall, Uma Maheswari, Michael Nuhn, Chuang Kee Ong, Bert Overduin, Michael Paulini, Helder Pedro, Emily Perry, Giulietta Spudich, Electra Tapanari, Brandon Walts, Gareth Williams, Marcela Tello–Ruiz, Joshua Stein, Sharon Wei, Doreen Ware, Daniel M. Bolser, Kevin L. Howe, Eugene Kulesha, Daniel Lawson, Gareth Maslen, Daniel M. Staines

**Affiliations:** 1 The European Molecular Biology Laboratory, The European Bioinformatics Institute, The Wellcome Genome Campus, Hinxton, Cambridgeshire, CB10 1SD, UK; 2 Cold Spring Harbor Laboratory, 1 Bungtown Road, Cold Spring Harbor, NY 11724, USA; 3 USDA-ARS NAA Plant, Soil and Nutrition Laboratory Research Unit, Cornell University, Ithaca, NY 14853, USA

## Abstract

Ensembl Genomes (http://www.ensemblgenomes.org) is an integrating resource for genome-scale data from non-vertebrate species, complementing the resources for vertebrate genomics developed in the context of the Ensembl project (http://www.ensembl.org). Together, the two resources provide a consistent set of programmatic and interactive interfaces to a rich range of data including reference sequence, gene models, transcriptional data, genetic variation and comparative analysis. This paper provides an update to the previous publications about the resource, with a focus on recent developments. These include the development of new analyses and views to represent polyploid genomes (of which bread wheat is the primary exemplar); and the continued up-scaling of the resource, which now includes over 23 000 bacterial genomes, 400 fungal genomes and 100 protist genomes, in addition to 55 genomes from invertebrate metazoa and 39 genomes from plants. This dramatic increase in the number of included genomes is one part of a broader effort to automate the integration of archival data (genome sequence, but also associated RNA sequence data and variant calls) within the context of reference genomes and make it available through the Ensembl user interfaces.

## OVERVIEW AND ACCESS

Ensembl Genomes (http://www.ensemblgenomes.org) is organized as five sites, each focused on one of the traditional kingdoms of life: bacteria, protists, fungi, plants and (invertebrate) metazoa. Vertebrate metazoa are the focus of the Ensembl project (http://www.ensembl.org) (1); Ensembl Genomes provides a complementary set of interfaces for non-vertebrate species. Core data available for all species includes genome sequence and annotations of protein-coding and non-coding genes; additional data includes transcriptional data, genetic variation and comparative analysis. Interactive access is provided through a web interface providing genome browsing capabilities: users can scroll through a graphical representation of a DNA molecule at various levels of resolution, seeing the relative locations of features—including conceptual annotations (e.g. genes, SNP loci), sequence patterns (e.g. repeats) and experimental data (e.g. sequences and external sequence features mapped onto the genome) supporting the primary annotations. Functional information is provided through direct curation, import from the UniProt Knowledgebase (2) or imputation from protein sequence (using the classification tool InterProScan (3)). We provide much of the data available on each page in a variety of formats for download, and tools that process and visualize various types of user-generated data in the context of the reference sequence and annotation. DNA and protein-based sequence search are also available. Fully referenced documentation of the analytical approaches taken is available online, and an online helpdesk (helpdesk@ensemblgenomes.org) provides a rapid response to users' questions.

The data are stored in a set of MySQL databases using the same schemas as those in use for the Ensembl project. Direct access to these is provided through a public MySQL server (host: mysql.ebi.ac.uk port:4157 username: anonymous) and additionally through well-developed Perl and RESTful APIs that provide an object-oriented framework for working with genomic data. Database dumps and common datasets (e.g. DNA, RNA and protein sequence sets, and sequence alignments) can be directly downloaded in bulk via FTP (ftp://ftp.ensemblgenomes.org). Ensembl source code is available from GitHub (https://github.com/Ensembl) under an open-source licence.

Ensembl Genomes data is also made available through a series of data warehouses, optimized around common (gene- and variant-centric) queries, using the BioMart data warehousing system (4). The BioMart framework provides a series of interfaces, including web-based query building tools, accessible at each of the Ensembl Genomes eukaryotic portals and a variety of other interfaces for interactive and programmatic access. BioMarts are not currently available for Ensembl Bacteria.

Ensembl Genomes is released 4–5 times a year, in synchrony with releases of Ensembl, utilizing the same software as the corresponding Ensembl release. The overall suite of Ensembl Genomes interfaces mirrors the interfaces provided for vertebrate genomes in Ensembl, and allows users access to genomic data from across the tree of life in a consistent manner.

## INVERTEBRATES AND PLANTS

Ensembl Genomes has continued to grow in 2014 and 2015. In the last two years, six species have been added to Ensembl Metazoa, bringing the total number of species included to 55, and 11 species to Ensembl Plants, bringing the total number of included species to 39. The new invertebrate species are the mountain pine beetle (*Dendroctonus ponderosae*) (5), the Glanville fritillary butterfly (*Melitaea cinxia*) (6), the warty comb jelly (*Mnemiopsis leidyi*) (7), a parasitic nematode (*Onchocerca volvulus*, the causative agent of river blindness), the red fire ant (*Solenopsis invicta*) (8) and the Nevada dampwood termite (*Zootermopsis nevadensis*) (9). The new plant species comprise a primitive flowering shrub (*Amborella trichopda*) (10), cabbage (*Brassica oleracea*) (11), cocoa (*Theobroma cacao*) (12), a wild grass (*Leersia perrieri*) (13), six species of rice (*Oryza barthii, Oryza glumaepatula*, *Oryza meridionalis*, *Oryza nivara*, *Oryza punctata* and *Oryza rufipogon*) (13) and bread wheat (*Triticum aestivum*) (14–16), bringing the total number of species represented to 39.

In addition, an ongoing process of data update continues for all genomes included in the database. In the same period, eight metazoan and eight plant genome assembly updates have occurred; and additionally, 14 new metazoan gene sets and two new plant gene sets have been released, annotated on existing assemblies. The plant databases are maintained jointly with the Gramene resource (http://www.gramene.org) (17) and can be accessed from either site.

## COMRPEHENSIVE COVERAGE OF MICRO-ORGANISMS

In Ensembl Genomes, genome sequence and annotation are taken directly from experts or databases recognized as authorities in their communities, where such resources exist; otherwise, the raw data is imported from the appropriate sequence archives and derived data are calculated as part of the Ensembl Genomes release process. Revisions to genome assemblies require re-alignment of any sequences that have been assigned a location on the genome by computation and a re-call of features (gene calls, variant calls, synteny blocks) that have been derived from such alignments. Updates to gene models require the assignment of new functional annotation even when the underlying assembly has not changed; and moreover, changes in just one species require the recalculation of all downstream comparative analyses. Genomes that are major foci of scientific research have mostly already been included in the resource; but genome sequence is increasingly available for a much larger number of species of interest to only small research communities, or which are of interest primarily in the context of comparative analysis. However, the cost (in terms of human and computer time) of importing, updating and calculating derived data (especially comparative data) has hitherto limited our the rate of growth of the resource and our ability to serve smaller communities.

Previously, we reported (18) the introduction of a new procedure to automatically update Ensembl Bacteria with all annotated genome sequence present in the archives of the International Nucleotide Sequence Database Collaboration (19). In addition to the data imported, basic functional annotation is added and a selection of species included in a broad-range comparative analysis. Since this report, we have continued to operate this pipeline and the number of bacterial species represented in the database has increased from ∼9000 to over 29 000. The same pipeline has since been applied to Ensembl Fungi and Ensembl Protists, increasing the number of represented fungal genomes to 408 (an eight-fold increase over the number previously included) and protist genomes to 133 (a fourfold increase). Associated revisions to the Ensembl interfaces and API have been introduced to support navigation and selection of genomes (following the model previously established for Ensembl Bacteria). With each release, gene models are automatically updated with new functional annotation: protein domains and gene functions defined using InterProScan (3) and the Gene Ontology (20). Additionally, one representative genome from every species (i.e. 273 fungal genomes and 89 protist genomes) are included in a comparative analysis (the Compara Gene Tree analysis (21)) with other genomes from the same kingdom. This generates evolutionary histories of every gene family and infers true orthologues by reconciliation with the species tree, and is updated with each release as new data becomes available.

## ALIGNMENTS AND VARIANTS

Closely related eukaryotic species are identified as suitable subjects for pairwise whole genome alignment, normally carried out using the lastZ (22) alignment tool followed by chaining and netting (23). For some groups of species, a well annotated genome is used as the point of reference for related species; in other cases, particularly where the genome is smaller, an all-versus-all approach is used. The number of pairwise alignments present in the database has increased to 1205 over the past two years. In addition, variation data are available for 24 species: new data incorporated since 2013 includes data from a new SNP-chip recently developed for the mosquito *Aedes aegytpi* (24), various datasets for barley (25–27) and wheat (see below), and resequencing data from 84 varieties of the tomato *Solanum lycopersicum* (28). Finally, alignments of gene expression (EST and RNA-seq) data are available for a total of 82 species. Users can additionally upload any positional data of their own or visualize data held locally in most common file formats (BAM, VCF, GFF, (Big)Wig, (Big)BED, etc.).

## FROM DIPLOIDY TO POLYPLOIDY

**Figure 1.**
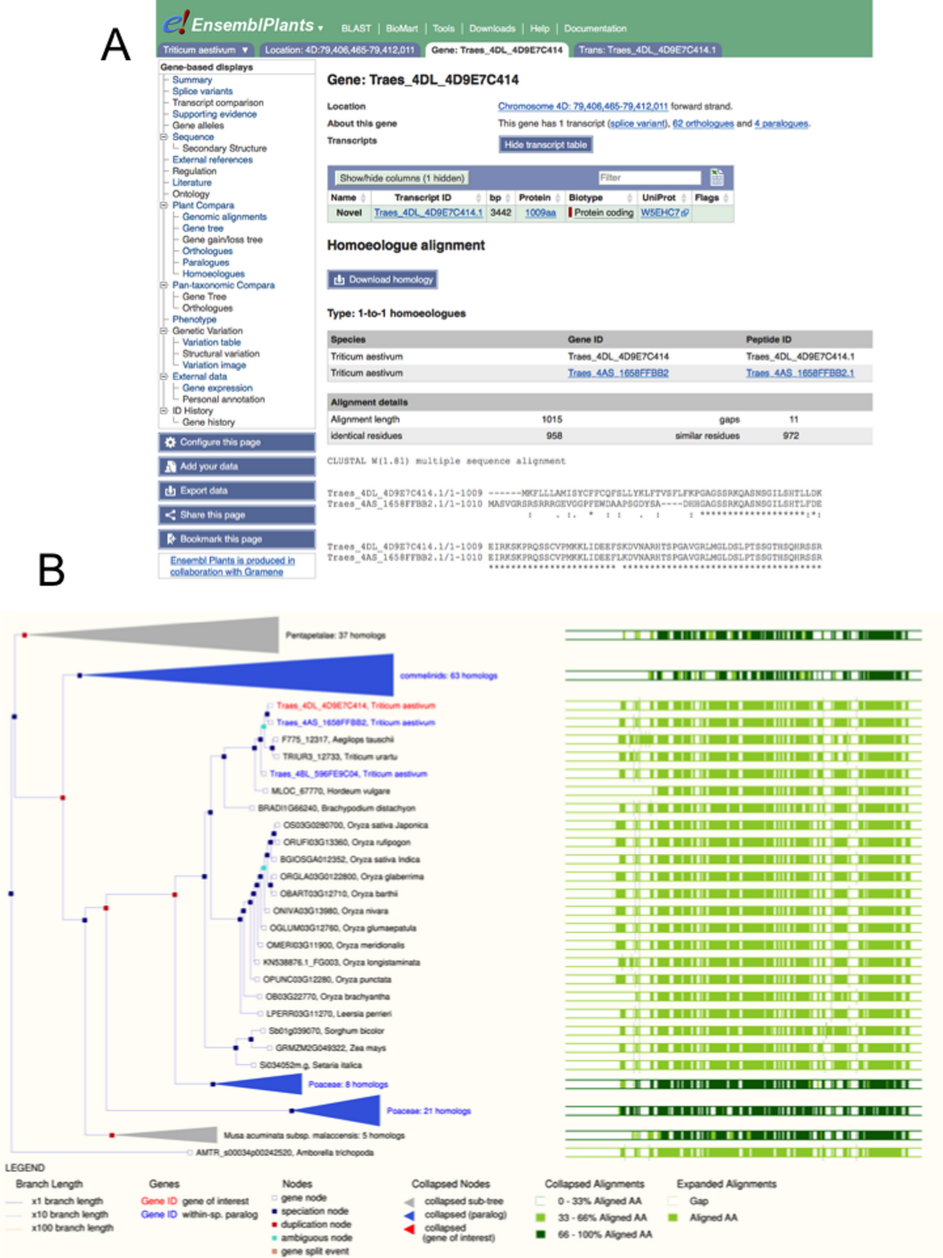
Comparative genomics of bread wheat, as visualized in Ensembl Plants. Panel **A** shows the alignments of two homoeologous genes at the level of protein sequence. The selected gene is highlighted in red. Panel **B** shows these genes in the wider context of a gene tree, showing 1:1 orthology over 21 grass genomes including the 3 bread wheat genomes and the two sequenced diploid precursors.

**Figure 2.**
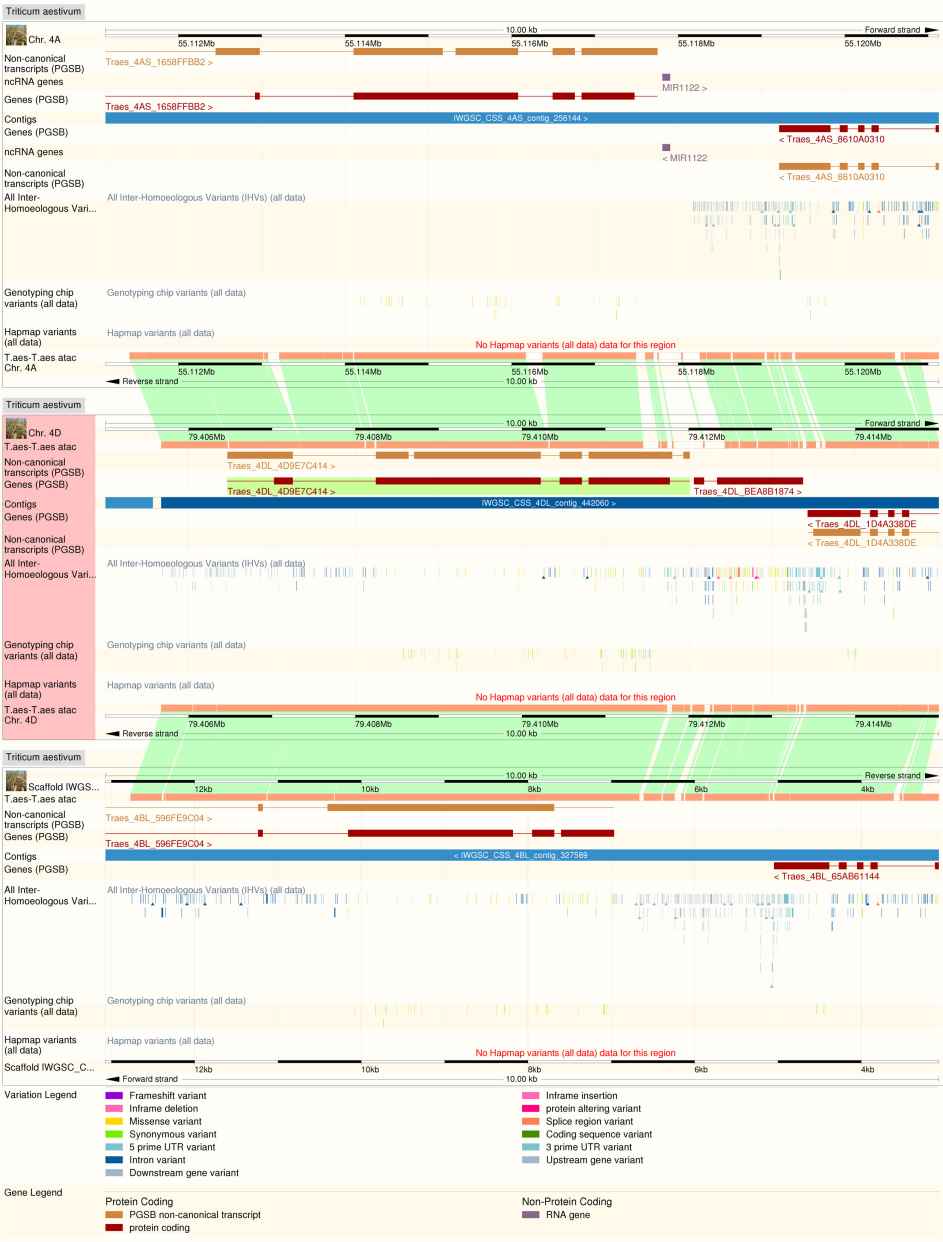
Whole genome alignment between the three bread wheat component genomes at a set of homoeologous loci. Inter-homoeologous variant calls and inter-variety polymorphisms are visible on tracks on each genome.

The recent release of genome sequence for the hexaploid bread wheat *Triticum aestivum* has been accommodated in Ensembl Plants with extensions to the analysis pipelines and user interfaces presented. The bread wheat genome is over five times larger than a human genome and while the best genome assembly is still fragmented, rapid incremental improvements have been released in recent years: Ensembl Plants has successively incorporated the data of Brenchley *el al*. (29); the International Wheat Genome Sequence Consortium's Chromosome Survey Sequence (14); and currently, an improved version of the latter enhanced by improved genetic mapping data (15) and a higher-quality assembly of the 3B chromosome (16). The large size of the wheat genome is partly due to its allohexaploidy, as it comprises of diploid genomes derived from three closely-related precursor species. Genome assemblies for two of these three precursors (*Triticum uratu*, the precursor of the bread wheat ‘A’ genome and *Aegilops tauschii*, the precursor of the ‘D’ genome), are also available in Ensembl Plants (the closest ancestor of the ‘B’ genome has not yet been unambiguously determined). To present the hexaploid in Ensembl Plants, alignments of the A, B and D genomes against each other have been generated, and which can be visualized in a pre-configured view. Additionally, in the gene tree analysis, the three wheat genomes are treated as separate species, allowing the evolutionary relationship of the genes from the different component genomes to be determined. The gene tree view has been linked into the genome alignment view via a new page, specifically presenting the ‘homoeologues’ (orthologues within the same species; see Figure 1) and the supporting evidence for the assessment (see Figure 2). Whole genome alignments between the bread wheat genomes and their diploid precursors, and also alignments to the genomes of other related species such as barely, *Brachypodium distachyon*, and rice, are also available.

Bread wheat belongs to the *Pooideae* subfamily of the *Poaceae* (the true grasses) and many important crop plants belong to this particular section of the taxonomy, which has evolved over a relatively short interval of 4 million years. We have prioritized *Pooideae* data for inclusion and the sub-family is now represented in the database by 21 distinct genomes (counting the A, B and D genomes of the hexaploid bread wheat separately), all which are included in the gene tree analysis for plants. Even though some of the presently available assemblies are still in a highly fragmented state, the assembly and annotation of the coding regions is reasonably complete and consistent; and a total of 918 gene families have been computationally identified with a single orthologue in every species and an inferred gene history exactly conformant with the taxonomy (Figure 1B). As more genomes are sequenced, and as the quality of available genome assemblies improves, it is to be expected that gene trees will offer increasingly accurate and comprehensive representations of evolutionary history, and that departures from the taxonomy will likely represent actual gene duplication or loss events and not artifacts of misannotation or misassembly.

The identification of homoeologues has in turn allowed the identification of inter-homoeologous variants—single nucleotide (and larger) variations between the A, B and D genomes. These are not necessarily polymorphisms as they may have become fixed since the ancestor species diverged. These data have been identified from the whole genome alignments in regions of 1:1 homoeology, and can be visualized alongside the inter-varietal polymorphisms also contained in the resource, which are imported from CerealsDB (30) and the wheat HapMap project (31).

Although bread wheat is the first polyploid species in Ensembl, common crop varieties of the *Brassica* genus are similarly allotetraploid, and two diploid precursors of the tetraploid species are already included in Ensembl Plants. It is therefore likely that the data structure and visualization interface developed for wheat will be deployed for further species in the near future.

## COMMUNITY AND COLLABORATION

Direct data curation by the scientific community has several potential benefits: people are likely to volunteer where the data is relevant to their own speciality, and thus in areas where their expertise is high and a research programme is active. Ensembl Genomes is working to encourage community-led curation in the context of our partnerships with WormBase (32), VectorBase (33), PhytoPath (Pedro *et al*., in press) /PHI-base (34) and PomBase (35), providing tools such as Web Apollo (36) and Canto (37) to allow the remote submission of structural and functional annotation. Through these collaborations, we have accommodated substantial community annotation of gene models for the parasitic worm *Brugia malayi*, seven species of invertebrate vectors and are currently collecting data from three fungal species; while community-derived functional annotations have been collected for *Schizosaccharomyces pombe* and numerous fungal phytopathogen species. New gene models curated through Web Apollo can be immediately visualized as a track in the Ensembl Genomes browsers, and are incrementally imported into the primary gene set. An automatic quality control process is applied which compares new community-supplied annotations to their predecessors, after which they are either accepted or (in the case of major discordance) sent for prior manual inspection before incorporation. The procedure also allows for the re-application of earlier manual curation following automatic re-annotation, ensuring that expert-supplied knowledge is not lost following subsequent analyses.

Collaboration with WormBase has also resulted in a new sister project, WormBase ParaSite (http://parasite.wormbase.org/) which provides access to 99 genomes from parasitic helminths through a compatible set of Ensembl interfaces (including web browser, BioMart and RESTful API). This model, of specialized sites with a focus on specific domains linked to Ensembl and Ensembl Genomes through integrated search and comparative genomics, is likely to become more common as certain domains of life are subject to increasingly comprehensive sequencing.

## FUTURE PERSPECTIVES: AUTOMATED ACCESS TO ARCHIVAL DATA

With the increasing volumes of available data in future, it is unlikely that most genome sequences will be subject to manual curation or quality control. For genomes where an insufficiently large community exists to sustain such activities, the Ensembl framework can still play a useful role, organizing both primary data and derived annotations through a standard interfaces in the context of reference genome sequence. A large amount of such data (reads, alignments, feature calls) has already been manually identified and made visible through Ensembl Genomes; but we are developing new pipelines to automatically identify (and, where necessary, align) RNA-seq and variant call data from the relevant archives (e.g. European Nucleotide Archive (38), European Variant Archive (http://www.ebi.ac.uk/eva)) and make these automatically accessible through Ensembl. Doing this successfully will require standards and support for the submission of appropriate meta data (sample and experimental descriptions) and the development of new interfaces within Ensembl to help users identify and select data for inclusion (based on the meta data attached). It is likely that track hubs (39) (a data format proposed by the UCSC Genome Browser and now implemented in Ensembl) will be used as the vehicle to deliver (potentially complex) data sets into the browser on demand; programatic retrieval of specified data sets will also be of growing importance as the number of genomes and alignments grow.
